# Osteogenesis imperfecta: a registry-based study of the clinical symptoms of disease in a large cohort of Italian patients

**DOI:** 10.3389/fendo.2026.1823717

**Published:** 2026-05-18

**Authors:** Marina Mordenti, James Clancy, Matthew Dyer, Manila Boarini, Federico Banchelli, Clive Whitcher, Marcella Lanza, Luca Sangiorgi

**Affiliations:** 1 Department of Rare Skeletal Disorders, IRCCS Istituto Ortopedico Rizzoli, Bologna, Italy; 2 Mereo BioPharma Group Plc, London, United Kingdom; 3 Independent researcher, Bologna, Italy

**Keywords:** bone fragility, brittle bone disease, cohort study, disease manifestations, disease registry, longitudinal data, osteogenesis imperfecta

## Abstract

**Introduction:**

Osteogenesis imperfecta (OI) is a rare, heritable, skeletal disorder resulting in defective bone formation. Clinical presentation is heterogeneous. This study aims to provide an overview of the clinical manifestations by disease severity and age in a large cohort of Italian patients with OI.

**Methods:**

Patients with OI who received care at the IRCCS Istituto Ortopedico Rizzoli from 2005 to 2024 were enrolled in the Registry of Osteogenesis Imperfecta database and analyzed for this study.

**Results:**

Overall, 628 patients with OI were included in the study (51.1% had Type I, 5.1% Type III, 6.7% Type IV, 0.3% Type V, and 36.8% unknown type). Patient height was reduced with increasing disease severity (Type I had mildly reduced height and Type III had marked height reduction). Fractures were common across all OI groups. The annualized fracture rates were highest in younger patients (288.31 per 1,000 person years) and patients with Type III, Type IV OI and unknown type. Skeletal deformities were present across all OI types and in every age group, with trunk deformities being most frequently reported. An estimated 10–30% of all patients suffered from functional limitations. Extraskeletal manifestations (skin abnormalities, hearing loss, valvulopathies, scleral discoloration, joint hyperlaxity, and dental abnormalities) were common.

**Conclusion:**

Various parameters including height, weight, fractures, and skeletal and extraskeletal deformities vary by OI type and patient age. These findings support the need for phenotype-informed, life-course care planning with the need for adaptations for age-related patterns to optimize patient care.

## 1 Introduction

Osteogenesis imperfecta (OI – ORPHA:666, ORPHA:216796, ORPHA:216812, ORPHA:216820, and ORPHA:216828), also known as brittle bone disease, is a rare, genetic, skeletal disorder, which occurs in 5 to 10 in 100,000 live births (1). OI is also now referred to as a collagen-related disorder, and is caused by defects in collagen structure, collagen folding, post-translational modification and processing, bone mineralization, and osteoblast differentiation, and results in defective bone formation (2, 3). This disease is both complex and heterogeneous, and is attributed to pathogenic variants in more than 20 different genes (4). Patients with OI present with a range of clinical symptoms including recurrent fractures, skeletal deformities and short stature, in addition to various extraskeletal manifestations, such as hearing loss, colored sclerae, and dental issues (including dentinogenesis imperfecta [DI]) (5).

Patients are typically classified according to clinical type/severity based on the Sillence classification system, with Types I–V being the most frequently diagnosed (6). The most common and least severe type of OI is Type I, with clinical presentations of bone fragility and blue sclerae. Type II is the most severe form of OI and is often lethal perinatally or shortly after birth. After Type II, Type III is the next most severe type of OI with clinical presentations of multiple fractures, progressive bone deformities, and DI. Patients with Type IV present with progressive deformities of the limbs and spine. Type V OI patients typically have interosseous ossification and may develop hyperplastic callus later in life (7–9). Due to disease heterogeneity and the complexities in OI presentation, patients require management by multidisciplinary teams.

One of the key challenges with rare diseases like OI, however, is access to treatments. Unfortunately, treatments for rare diseases face many hurdles including limited patient populations, clinical trial endpoints, and the evidence needed for regulatory review and approval, as well as the use of small datasets to demonstrate compelling cost-benefit to support positive pricing and reimbursement (10). To address this, the Food and Drug Administration (FDA) and European Medicines Agency (EMA) have outlined that the use of real-world data and real-world evidence should be considered as a potential source of data to help address the challenges facing treatments for rare diseases (11, 12). In OI, a project has been established to create a common core dataset using existing, well-established data sources to meet the needs of multiple stakeholders (10, 13). The SATURN (**S**ystematic **A**ccumulation of **T**reatment practices and **U**tilisation, **R**eal-world evidence and **N**atural history data) program is funded by Mereo BioPharma with collaboration from the Osteogenesis Imperfecta Federation Europe (OIFE), the European Reference Network on Rare Bone Diseases (ERN BOND https://ernbond.eu/), and IRCCS Istituto Ortopedico Rizzoli (IOR).

In 2024, we published the first analysis from Registry of Osteogenesis Imperfecta (ROI) database which, at that time, included 568 patients with OI. That cross-sectional study presented the skeletal and extraskeletal features of patients with OI in this large cohort of Italian patients (14). Since that publication, the OI population in the registry has increased. The aim of this manuscript is to investigate further the age- and OI type-related patterns of OI manifestations in this expanded, well-characterized cohort of patients with OI from the IOR, and to include novel data on fractures during a long-term follow-up and on the evolution of patient height.

## 2 Materials and methods

### 2.1 Study design and the ROI database

This study was an observational, non-interventional, retrospective, registry-based cohort study using data collected within the Italian ROI (NCT04115774) framework. The study was approved by the Area Vasta Emilia Centro Ethics Committee (CE AVEC) in August 2023 (455/2023/Oss/IOR).

Patients were included in the analysis if they were at least 2 years old, with a confirmed or suspected diagnosis of OI, with or without a known clinical OI type. Patients were classified with unknown OI type if they were in the early stages of disease presentation (e.g., very young children) or if they had clinical manifestations that could be attributed to more than one OI type (e.g., OI types III and IV can have similar characteristics). Patients <2 years of age were excluded because their symptoms were usually mild and a definitive diagnosis of OI could not be confirmed. Patients were also excluded from the analysis if a differential diagnosis between suspected OI and another musculoskeletal rare disease was recorded. Data was captured from electronic health records, patient reports, and medical files and stored in a GDPR-compliant platform, serving as the basis for the ROI.

The baseline enrolment date was the date of the first visit recorded in the ROI database between January 2005 and December 2024. Patient follow-up data was available if the patient had had at least one subsequent visit that was more than one year from their baseline visit, with data recorded in the ROI database, with a cut-off of December 2024.

All the data included in the manuscript are based on electronic health records, imaging, clinical reports data. Skeletal manifestations, including fractures, were assessed by orthopaedic surgeons with long-standing expertise in OI, while extraskeletal manifestations were evaluated by expert geneticists.

### 2.2 Statistical analyses

The quantitative variables were described as the mean, median, standard deviation (SD), and interquartile range (IQR), whereas the qualitative variables were presented as numbers and percentages. Analyses were stratified by OI type (I, III, IV, V, or unknown) and age class at enrolment (2–17 years, 18–49 years, and ≥50 years). Analyses for the annualized evolution of height per patient among OI patients with follow-up data aged ≤17 years were stratified by 2–9 years and 10–17 years age classes. Normalized z-scores of anthropometric measurements, such as height and weight, were calculated using age- and sex-matched reference charts for the Italian population aged 2–20 years (15). For patients aged >20 years, the reference charts for 20-year-old subjects were used. The prevalence of skeletal and extraskeletal OI features, as well as deformities and functional limitations at baseline, was reported.

The length of follow-up was measured as the time span in person-years between the date of enrolment and the date of the last visit in the ROI database (where available). The fracture rate over time, overall and by fracture location, was measured as the ratio between the total number of fractures and the sum of person-years over patients. The association between OI type, age at enrolment, gender and the fracture rate was assessed with unadjusted and adjusted multivariable Poisson models, and results were expressed as the incidence rate ratio (IRR). The variation in height and its z-scores over time were calculated as the difference between the last and first recorded values, divided by the time span in person-years between the two measurements. Information on fractures and height during follow up were derived from visits after enrolment. Uncertainty around mean estimates of prevalence, rate and rate ratio measures was described with 95% confidence intervals (CI). CIs for prevalence measures were calculated with the Clopper-Pearson exact method, whereas for rate measures with the Pearson exact method. Statistical significance was set at p<0.05. Missing values, when present, were presented in tables. Data management and statistical analysis were carried out with R 4.3.1 software (The R Foundation for Statistical Computing, Wien).

## 3 Results

### 3.1 Patient demographics at baseline and length of follow-up

A total of 628 patients with OI were included in this analysis. Over half of subjects in this study had Type I OI (51.1%), 36.8% had an unknown type of OI, 6.7% of patients had Type IV, 5.1% had Type III, and 0.3% had Type V (**Table 1A**). Most patients were aged 2–9 years and 18–34 years at baseline (28.0% and 24.2%, respectively). The mean age at enrolment across all OI types was 24.49 years (SD 18.11); patients with Type V had a lower age of 16 years (SD 16.97) at enrolment. There were marginally more male patients (53.5%) than female patients (46.5%), except for Type III OI and unknown OI type, where there were marginally more female patients. Disease inheritance was almost equally distributed between positive (40%) and negative (38.9%). The predominant genetic cause for OI was mutations in the *COL1A1* gene, followed by *COL1A2* gene (**Supplementary Table 1**).

**Table 1A:** patient characteristics at baseline.

Variable	Type I	Type III	Type IV	Type V	Unknown	Total
Age (N = 628)						
2–9 years, n (%)	92 (14.6)	11 (1.8)	8 (1.3)	1 (0.2)	64 (10.2)	176 (28.0)
10–17 years, n (%)	53 (8.4)	1 (0.2)	5 (0.8)	0 (0.0)	52 (8.3)	111 (17.7)
18–34 years, n (%)	82 (13.1)	9 (1.4)	20 (3.2)	1 (0.2)	40 (6.4)	152 (24.2)
35–49 years, n (%)	61 (9.7)	8 (1.3)	5 (0.8)	0 (0.0)	47 (7.5)	121 (19.3)
≥50 years, n (%)	33 (5.3)	3 (0.5)	4 (0.6)	0 (0.0)	28 (4.5)	68 (10.8)
Total, n (%)	321 (51.1)	32 (5.1)	42 (6.7)	2 (0.3)	231 (36.8)	628 (100)
Age at enrolment, mean (SD)	24.41 (17.91)	24.81 (18.02)	25.10 (14.80)	16.00 (16.97)	24.45 (18.12)	24.49 (18.11)
Age at enrolment, median (IQR)	23 (8, 36)	27 (8, 38)	23 (14, 32)	16 (10, 22)	17 (8, 37)	22 (8, 37)
Gender						
Male, n (%)	184 (29.3)	14 (2.2)	25 (4.0)	1 (0.2)	112 (17.8)	336 (53.5)
Female, n (%)	137 (21.8)	18 (2.9)	17 (2.7)	1 (0.2)	119 (18.5)	292 (46.5)
Year of enrolment (N = 628)						
2005–2009, n (%)	76 (12.1)	11 (1.8)	12 (1.9)	1 (0.2)	21 (3.3)	121 (19.3)
2010–2014, n (%)	140 (22.3)	16 (2.5)	23 (3.7)	1 (0.2)	67 (10.7)	247 (39.9)
2015–2019, n (%)	84 (13.4)	4 (0.6)	7 (1.1)	0 (0.0)	81 (12.9)	176 (28.0)
2020–2024, n (%)	21 (3.3)	1 (0.2)	0 (0.0)	0 (0.0)	62 (9.9)	84 (13.4)
Height z-score (N = 546)*						
N	287	22	38	2	197	546
z-score, mean (SD)	-0.89 (1.33)	-7.16 (3.85)	-2.89 (2.63)	-2.74 (4.40)	-0.92 (1.69)	-1.29 (2.17)
z-score, median (IQR)	-0.88 (-1.86, -0.02)	-7.52 (-9.35, -4.76)	-2.55 (-4.16, -1.60)	-2.74 (-4.30, -1.19)	-0.63 (-1.81, 0.17)	-1.05 (-2.15, -0.02)
Weight z-score (N = 494)^†^						
N	265	24	36	2	167	494
z-score, mean (SD)	-0.22 (1.30)	-4.36 (3.51)	-1.18 (2.10)	-0.80 (1.20)	-0.45 (1.35)	-0.57 (1.79)
z-score, median (IQR)	-0.32 (-1.04, 0.57)	-4.29 (-6.60, -1.49)	-0.79 (-1.90, 0.20)	-0.80 (-1.22, -0.37)	-0.37 (-1.34, 0.53)	-0.38 (-1.32, 0.47)

IQR, interquartile range; SD, standard deviation.

*Data available for 546 patients. ^†^Data available for 494 patients.

**Table 1B:** Annualized evolution of height per patient among OI patients with follow-up data aged ≤17 years.

Variable	Type I	Type III	Type IV	Type V	Unknown	Total
Age (N = 42)						
2–9 years, n (%)	16 (38.1)	1 (2.4)	0 (0.0)	0 (0.0)	10 (23.8)	27 (64.3)
10–17 years, n (%)	6 (14.3)	0 (0.0)	0 (0.0)	0 (0.0)	9 (21.4)	15 (35.7)
Total	22 (52.4)	1 (2.4)	0 (0.0)	0 (0.0)	19 (45.2)	42 (100)
Height z-score evolution (N = 42)						
z-score at first measurement Mean (SD) Median (IQR)	-0.36 (1.48) -0.21 (-1.34, 0.63)	0.03 (NC) 0.28 (0.28, 0.28)	0 0	0 0	-0.47 (1.41) -0.65 (-1.30, 0.78)	-0.40 (1.47) -0.29 (-1.34, 0.71)
z-score at last measurement Mean (SD) Median (IQR)	-0.19 (1.15) -0.02 (-1.11, 0.81)	-0.11 (NC) -0.11 (-0.11, -0.11)	0 0	0 0	-0.40 (1.83) -0.34 (-0.92, 0.90)	-0.28 (1.47) -0.09 (-0.97, 0.81)
Annualized evolution of height z-score per patient Mean (SD) Median (IQR)	0.05 (0.39) -0.11 (-0.13, 0.08)	-0.06 (NC) -0.06 (-0.06, -0.06)	0 0	0 0	0.10 (0.33) 0.06 (-0.14, 0.19)	-0.04 (0.47) 0.01 (-0.15, 0.19)

IQR, interquartile range; NC, not calculated due to availability of data for only one patient; SD, standard deviation.

At baseline, patients with Type I OI and unknown type of OI were slightly shorter than average (mean z-scores -0.89 [SD 1.33] and -0.92 [1.69], respectively, compared to reference charts for the Italian population (15)), while patients with Types IV and V had more reduced height (-2.89 [2.63] and -2.74 [4.40], respectively), and patients with Type III OI had substantially reduced height (-7.16 [3.85]). Patient weight had a similar trend to patient height, with below average weight being lower in patients with Type I and unknown type and lowest in patients with Type III OI (-0.22 [1.30] and -0.45 [1.35] vs -4.36 [3.51], respectively). Follow-up data was available for 170 patients out of the 628 included. The follow-up time ranged from 1.01 to 17.68 years, with an average was 5.8 years (7.03 years for Type I OI, 6.46 years for Type III, 6.96 years for Type IV, 1.69 years for Type V, and 3.86 years for unknown type OI).

### 3.2 Annualized evolution of height

The annualized evolution of height was measured in 42 pediatric patients (between the ages of 2 and 17 years) (**Table 1B**). Of these patients, 22 (52.4%) had Type I OI, 1 (2.4%) had Type III, and 19 (45.2%) had an unknown type of OI. Patients with Type I and unknown type OI tended to be shorter in stature than the healthy reference population, as shown by the negative z-scores at first and last measurement (first visit mean [SD] -0.36 [1.48] and -0.47 [1.41], respectively, and last visit -0.19 [1.15] and -0.40 [1.83], respectively); the patient with Type III OI also appeared shorter in stature at their last measurement (-0.11, SD not applicable). The annualized evolution of height z-scores was close to zero for all patients suggesting that their growth pattern was stable (mean [SD] 0.05 for Type I 0.05 [0.39], -0.06 for Type III and 0.10 [0.33] for unknown type).

### 3.3 Annualized fracture rate for patients with OI

Overall, 170 patients were included in the annualized fracture rate calculations with most patients having either Type I (n=84) or unknown type (n=63) OI (**Table 2**). Of these patients, 76 (44.7%) had at least one fracture during the follow-up period, with 42 (50.0%) and 23 (36.5%) patients with Type I and unknown type OI, respectively, having at least one fracture. Patients reported a mean number of 1.20 (SD 1.95) fractures during the entire follow-up period, with 1.26 (SD 1.88), 1.38 (SD (2.33), 1.79 (SD 1.93), and 0.98 (SD 2.02) fractures per patient occurring in patients with Type I, Type III, Type IV, and unknown type OI, respectively. Overall, 41.8% of patients aged 2–17 years had fractures during follow-up, with a higher percentage of patients with Type I and unknown type OI having fractures (39.2% and 52.4%, respectively). Between 18 and 49 years of age, 46.5% of patients had fractures, with higher percentages being seen in patients with Type I and Type IV (51.2% and 71.4%, respectively). Fewer fractures occurred in patients aged ≥50 years (11.8% for all patients).

**Table 2A:** Annualized number of fractures among OI patients with follow-up data.

Variable	Type I	Type III	Type IV	Type V	Unknown	Total
Number of fractures reported during the follow-up period by age (N = 170)						
2–17 years, n (%)	33 (39.2)	2 (25.0)	3 (21.4)	0 (0.0)	33 (52.4)	71 (41.8)
18–49 years, n (%)	43 (51.2)	3 (37.5)	10 (71.4)	1 (100)	22 (34.9)	79 (46.5)
≥50 years, n (%)	8 (9.5)	3 (37.5)	1 (7.1)	0 (0.0)	8 (12.7)	20 (11.8)
Total	84 (100)	8 (100)	14 (100)	1 (100)	63 (100)	170 (100)
Duration of follow-up (time between first and last visit in the database [years])						
Mean (SD)	7.03 (4.84)	6.55 (5.23)	6.96 (4.15)	1.69 (NC)	3.86 (3.51)	5.80 (4.57)
Median (IQR)	5.62 (2.72, 11.51)	6.01 (1.76, 10.52)	7.02 (3.72, 9.79)	1.69 (1.69, 1.69)	2.54 (1.46, 4.56)	3.67 (2.01, 9.05)
Number of visits in the database, n						
Mean (SD)	4.45 (3.13)	6.38 (6.65)	5.79 (4.28)	2.00 (NA)	4.56 (5.17)	4.67 (4.26)
Median (IQR)	3.00 (2.00, 6.00)	3.00 (2.00, 7.75)	4.00 (2.00, 7.75)	2.00 (2.00, 2.00)	3.00 (2.00, 4.00)	3.00 (3.00, 5.75)
Delay between initial visit and first subsequent visit in the database (years)						
Mean (SD)	3.37 (4.11)	1.35 (0.98)	2.38 (2.52)	1.69 (NA)	1.84 (2.37)	2.62 (3.39)
Median (IQR)	1.32 (0.60, 4.73)	1.20 (0.88, 1.53)	1.29 (0.56, 3.51)	1.69 (1.69, 1.69)	1.11 (0.57, 2.12)	1.29 (0.57, 2.91)
At least one fracture during the entire FU period, n (%)	42 (50)	3 (37.5)	8 (57.1)	0 (0.0)	23 (36.5)	76 (44.7)
Number of fractures per patient reported during the entire FU period (N = 170)						
Mean (SD)	1.26 (1.88)	1.38 (2.33)	1.79 (1.93)	0	0.98 (2.02)	1.20 (1.95)
Median (IQR)	0.5 (0, 2)	0 (0, 1.75)	1.5 (0, 3)	0 (0, 0)	0 (0, 1)	0 (0, 2)
Annualized fracture rate in the population during the entire FU period (N = 170)						
N	84	8	14	1	63	170
N fractures	106	11	25	0	62	204
Person-years	590.66	52.36	97.41	1.69	242.98	985.10
Rate per 1000 PYs (95% CI)	179.46 (146.93, 217.05)	210.08 (104.87, 375.90)	256.64 (166.09, 378.86)	0.00 (NC)	255.17 (195.63, 327.11)	207.09 (179.64, 237.54)
Annualized fracture rate in the population during the entire FU period, by age group (N = 170)						
2–17 years						
N	33	2	3	0	33	71
N fractures	56	10	7	0	41	114
Person-years	242.49	21.11	12.35	0	119.46	395.41
Rate per 1000 PYs (95% CI)	230.93 (174.45, 299.89)	473.71 (227.16, 871.17)	566.80 (227.88, 1167.83)	NC	343.21 (246.29, 465.60)	288.31 (237.82, 346.35)
18–49 years						
N	43	3	10	1	22	79
N fractures	47	1	15	0	16	79
Person-years	319.59	24.74	75.22	1.69	81.26	502.49
Rate per 1000 PYs (95% CI)	147.06 (108.06, 195.56)	40.42 (1.02, 225.21)	199.42 (111.61, 328.90)	0.00 (NC)	196.90 (112.54, 319.75	157.22 (124.47, 195.94)
≥50 years						
N	8	3	1	0	8	20
N fractures	3	0	3	0	5	11
Person-years	28.59	6.50	9.85	0	42.27	87.20
Rate per 1000 PYs (95% CI)	104.93 (21.64, 306.66)	0.00 (NC)	304.57 (62.81, 890.08)	NC	118.29 (38.41, 276.04)	126.15 (62.97, 225.71)

CI, confidence interval; FU, follow-up; IQR, interquartile range; NC, not calculated due to absence of patients or fractures; PY, person-years; SD, standard deviation.

**Table 2B:** Unadjusted and adjusted multivariable analyses on annualized fracture rate during the entire follow-up period.

Comparison	Unadjusted analysis			Adjusted analysis*		
	IRR	95% CI	p	IRR	95% CI	p
Type III vs Type I	1.17	0.63–2.18	0.6189	1.19	0.63–2.26	0.5858
Type IV vs Type I	1.66	1.10–2.50	0.0157	2.05	1.34–3.13	0.0009
Type V vs Type I	NA	NA	NA	NA	NA	NA
Unknown Type vs Type I	1.42	1.04–1.95	0.0277	1.39	1.00–1.92	0.0483
Age 18–49 vs 2–17 years	0.57	0.43–0.76	0.0001	0.54	0.40–0.73	<0.0001
Age ≥50 vs 2–17 years	0.44	0.24–0.81	0.0088	0.38	0.21–0.72	0.0027
Gender M vs F	1.07	0.81–1.41	0.6238	1.00	0.75–1.34	0.9809

*Adjusted for OI type, age class at enrolment, and gender.

CI, confidence interval; F, female; IRR, incidence rate ratio; M, male; NA, not applicable due to absence of fractures during follow-up in Type V patients.

The annualized fracture rate was highest in patients aged 2–17 years – 230.93 (95% CI 174.45, 299.89), 473 (227.16, 871.17), 566.80 (227.88, 1167.83), and 343.21 (246.29, 465.60) per 1,000 person years for patients with Type I, Type III, Type IV, and unknown type OI, respectively (**Table 2A**). As age increased, the annualized fracture rate decreased and was lowest in patients aged ≥50 years, respectively.

**Figure 1:**
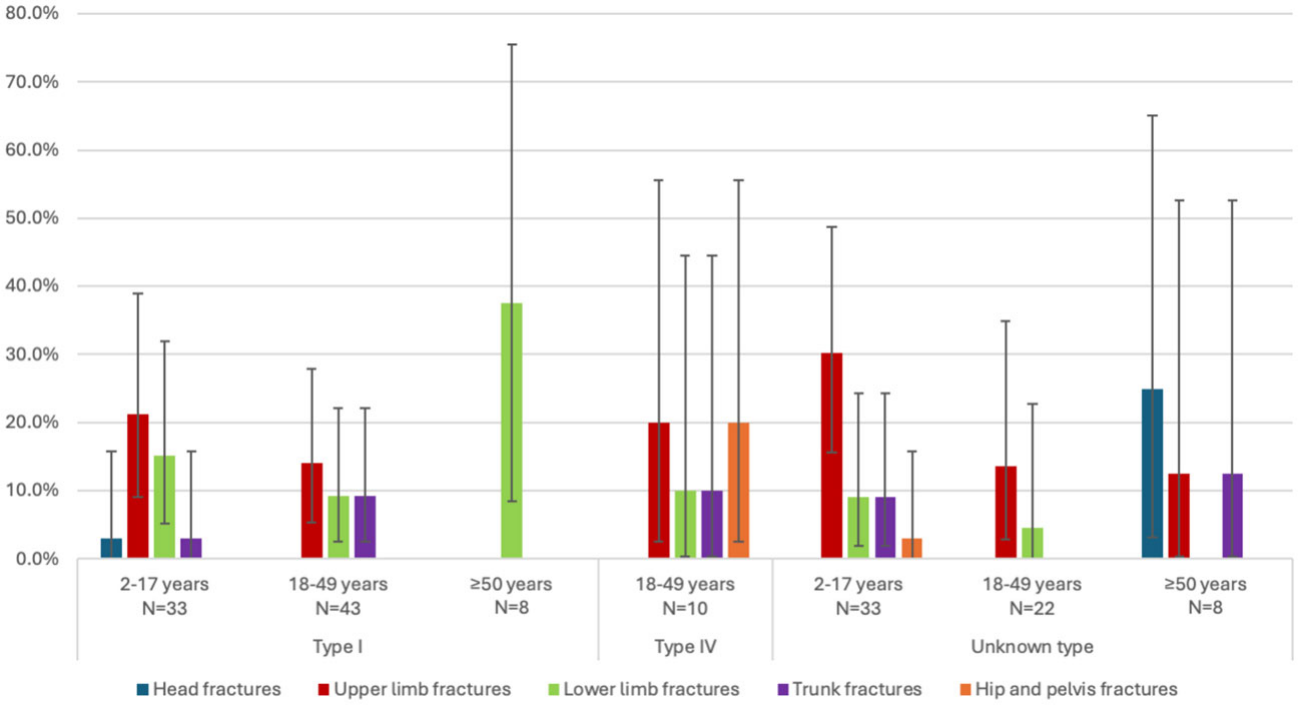
Location of fractures during the follow-up period by OI type and age group in N>5 patients/group. Patients may have more than one fracture type. Error bars represent the 95% confidence intervals. Confidence intervals were displayed only for prevalence measures >0% and <100%.

According to the multivariable adjusted analysis, fracture rate was higher in Type IV (IRR 2.05, p=0.0009) and unknown type (IRR 1.39, p=0.0483) patients compared to Type I, whereas the difference between Type III and Type I was not statistically significant (IRR 1.19, p=0.5858). Furthermore, patients aged 18–49 years (IRR 0.54, p<0.0001) and ≥50 years (IRR 0.38, p=0.0027) had a lower risk of fracture. There was no difference between males and females (**Table 2B**). The location of fractures by OI type is presented in **Figure 1**. Upper limb fractures were present across all OI types and were the most common types of fractures in patients with Type III, IV and unknown type OI. Patients with Type I, IV and unknown type OI also suffered from lower limb fractures. Hip and pelvis fractures tended to occur in the more severe OI types (Types III and IV). Head fractures were uncommon and only occurred in patients with Type I OI.

### 3.4 Skeletal manifestations

**Figure 2:**
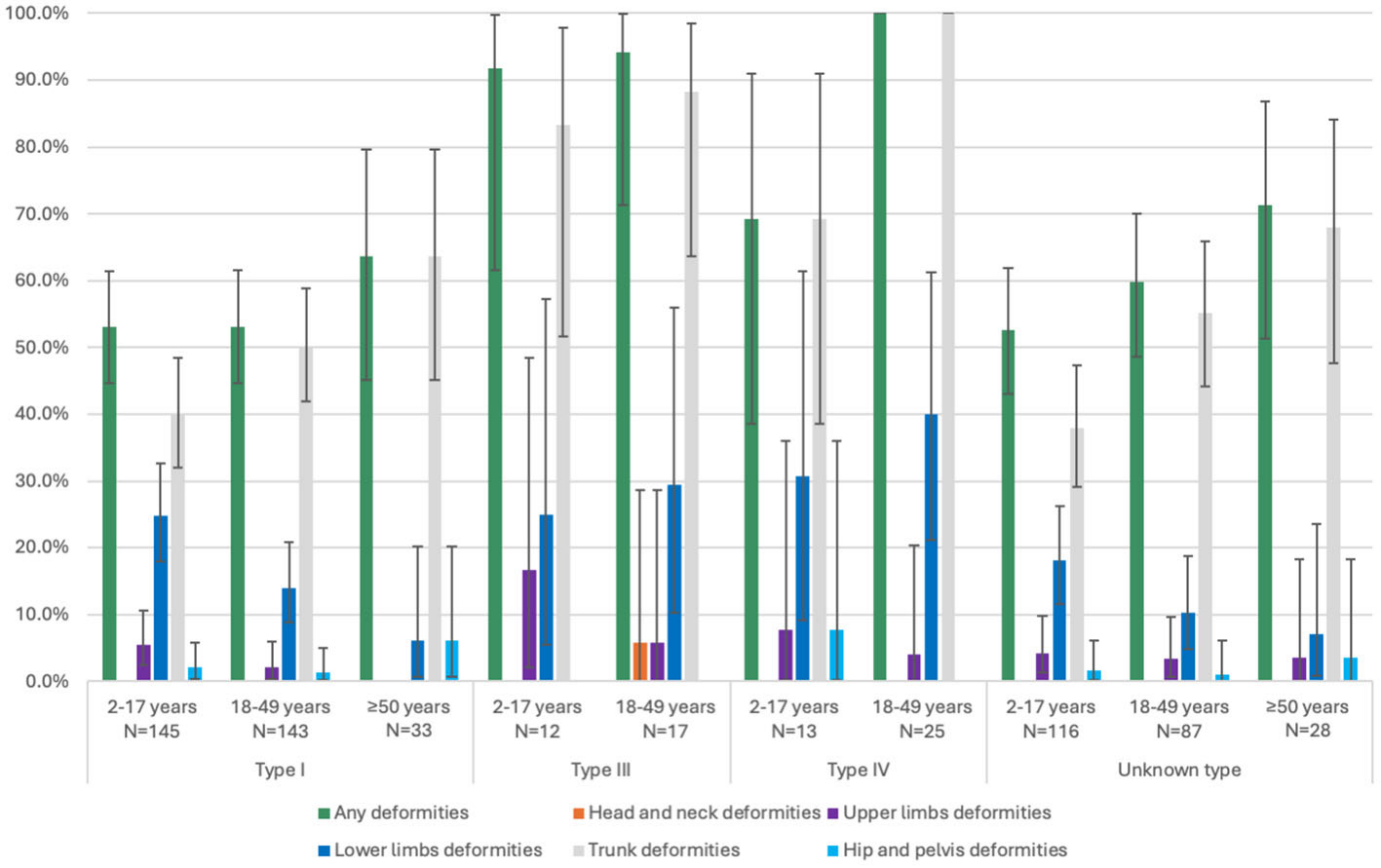
Prevalence of skeletal deformities at baseline by OI type and age group in N>5 patients/group. Patients may have more than one deformity type. Error bars represent the 95% confidence intervals. Confidence intervals were displayed only for prevalence measures >0% and <100%.

At baseline, skeletal deformities were present across all OI types and in every age group (**Figure 2**). Trunk deformities (which included spine alignment and rib cage deformities) were the most reported skeletal deformities, with higher frequences being observed in older age groups and in patients with Types III and IV OI. In Type I OI, the prevalence ranged from 37.9% in children aged 2–17 years to 100% in adults aged ≥50 years; in Type III OI, the prevalence was 83.3% in children, 88.2% in adults aged 18–49 years, and 100% in adults aged ≥50 years; in Type IV OI, the prevalence was 69.2% in children and 100% in adults aged 18–49 years; and in unknown type OI, the prevalence was 37.9% in children, 55.2% in adults aged 18–49 years, and 67.9% in adults aged ≥50 years. Lower limb deformities were common in the more severe OI types, affecting up to 40% of adults with Type IV OI, while upper limb deformities were less frequent (with a higher occurrence in all ages of patients with Type III OI). Head and neck deformities and hip and pelvis deformities were comparatively rare across all OI types. Longitudinal data for patients with at least two data points, at least one year apart, are presented in **Supplementary Figure 1**.

Almost half of patients suffered from bone densitometry abnormalities, which increased with age (**Supplementary Table 2**); the exceptions included patients with Type III OI where bone densitometry abnormalities decreased with increasing age and adolescent patients with unknown OI where only 27.6% showed bone densitometry abnormalities. Except for Type V OI, vertebral compression fractures occurred in all types of OI but were more common in Type IV and unknown type OI. Numbers of vertebral compression fractures typically increased with increasing age. Wormian bones were more common in younger patients (9.6%, 16.7%, 23.1%, and 2.6% of patients with Type I, III, IV, and unknown type OI). There were fewer cases of Wormian bones in patients aged 18–49 years and no cases in patients aged ≥50 years. Facial dysmorphism, which included triangular face, maxilla dysmorphia, frontal bossing, and other facial dysmorphisms, were more common in patients with Type III (33.3%, 58.8%, and 33.3% of patients aged 2–17, 18–49, and ≥50 years, respectively) and Type IV (38.5%, 44.0%, and 25.0% of patients aged 2–17, 18–49, and ≥50 years, respectively). An estimated 10–30% of patients suffered from functional limitations (which included limitations in the head and neck, upper limbs, lower limbs, trunk, and hip and pelvis).

### 3.5 Extraskeletal manifestations

A significant percentage of patients with OI had skin abnormalities, with no clear trend by OI type or age range (**Table 3**). These skin abnormalities included morphological skin abnormalities, cutis laxa, skin lesions, other skin abnormalities, with cutis laxa being the most common (**Supplementary Table 3**).

**Table 3:** Prevalence of extraskeletal manifestations by OI type and age group.

Extraskeletal manifestations	Skin abnormalities	Hearing loss	Valvulo-pathy	Scleral discoloration	Joint hyperlaxity	Dental abnormalities
Type I OI (N = 321)						
2–17 years (n=145), n (%) [95% CI]	25 (17.2) [11.5, 24.4]	16 (11.0) [6.4, 17.3]	12 (8.3) [4.3, 14.0]	126 (86.9) [80.3, 91.9]	70 (48.3) [39.9, 56.7]	14 (9.7) [5.4, 15.7]
18–49 years (n=143), n (%) [95% CI]	27 (18.9) [12.8, 26.3]	36 (25.2) [18.3, 33.1]	14 (9.8) [5.5, 15.9]	121 (84.6) [77.6, 90.1]	57 (39.9) [31.8, 48.4]	28 (19.6) [13.4, 27.0]
≥50 years (n=33), n (%) [95% CI]	9 (27.3) [13.3, 45.5]	18 (54.5) [36.4, 71.9]	5 (15.2) [5.1, 31.9]	25 (75.8) [57.7, 88.9]	11 (33.3) [18.0, 51.8]	9 (27.3) [13.3, 45.5]
Type III OI (N = 32)						
2–17 years (n=12), n (%) [95% CI]	3 (25.0) [5.5, 57.2]	1 (8.3) [0.2, 38.5]	1 (8.3) [0.2, 38.5]	6 (50.0) [21.1, 78.9]	4 (33.3) [9.9, 65.1]	3 (25.0) [5.5, 57.2]
18–49 years (n=17), n (%) [95% CI]	2 (11.8) [1.5, 36.4]	4 (23.5) [6.8, 49.9]	1 (5.9) [0.1, 28.7]	13 (76.5) [50.1, 93.2]	6 (35.3) [14.2, 61.7]	8 (47.1) [23.0, 72.2]
≥50 years (n=3), n (%) [95% CI]	0 (0.0) [0.0, 70.8]	3 (100.0) [29.2, 100.0]	1 (33.3) [0.8, 90.6]	2 (66.7) [9.4, 99.2]	0 (0.0) [0.0, 70.8]	1 (33.3) [0.8, 90.6]
Type IV OI (N = 42)						
2–17 years (n=13), n (%) [95% CI]	2 (15.4) [1.9, 45.4]	0 (0.0) [0.0, 24.7]	2 (15.4) [1.9, 45.4]	12 (92.3) [64.0, 100.0]	6 (46.2) [19.2, 74.9]	1 (7.7) [0.2, 36.0]
18–49 years (n=25), n (%) [95% CI]	3 (12.0) [2.5, 31.2]	7 (28.0) [12.1, 49.4]	6 (24.0) [9.4, 45.1]	20 (80.0) [59.3, 93.2]	14 (56.0) [34.9, 75.6]	11 (44.0) [24.4, 65.1]
≥50 years (n=4), n (%) [95% CI]	1 (25.0) [0.6, 80.6]	2 (50.0) [6.8, 93.2]	0 (0.0) [0.0, 60.2]	4 (100.0) [39.8, 100.0]	1 (25.0) [0.6, 80.6]	2 (50.0) [6.8, 93.2]
Type V OI (N = 2)						
2–17 years (n=1), n (%) [95% CI]	0 (0.0) [0.0, 97.5]	0 (0.0) [0.0, 97.5]	0 (0.0) [0.0, 97.5]	0 (0.0) [0.0, 97.5]	0 (0.0) [0.0, 97.5]	0 (0.0) [0.0, 97.5]
18–49 years (n=1), n (%) [95% CI]	1 (100.0) [2.5, 100.0]	0 (0.0) [0.0, 97.5]	0 (0.0) [0.0, 97.5]	0 (0.0) [0.0, 97.5]	0 (0.0) [0.0, 97.5]	0 (0.0) [0.0, 97.5]
≥50 years (n=0), n (%) [95% CI]	NC	NC	NC	NC	NC	NC
Unknown type OI (N = 231)						
2–17 years (n=116), n (%) [95% CI]	21 (18.1) [11.6, 26.3]	1 (0.9) [0.0, 4.7]	8 (6.9) [3.0, 13.1]	66 (56.9) [47.4, 66.1]	48 (41.4) [32.3, 50.9]	22 (19.0) [12.3, 27.3]
18–49 years (n=87), n (%) [95% CI]	8 (9.2) [4.1, 17.3]	15 (17.2) [10.0, 26.8]	8 (9.2) [4.1, 17.3]	45 (51.7) [40.8, 62.6]	27 (31.0) [21.5, 41.9]	19 (21.8) [13.7, 32.0]
≥50 years (n=28), n (%) [95% CI]	6 (21.4) [8.3, 41.0]	9 (32.1) [15.9, 52.4]	1 (3.6) [0.1, 18.3]	19 (67.9) [47.6, 84.1]	5 (17.9) [6.1, 36.9]	5 (17.9) [6.1, 36.9]

CI, confidence interval; NC, not calculated due to absence of patients.

Hearing loss, which included mixed hearing loss, conductive hearing loss, sensorineural hearing loss, and other types of hearing loss, occurred in all patient ages irrespective of OI type (except for patients with Type V OI, which could be attributed to the low numbers of subjects in this group) (**Supplementary Table 4**). Typically hearing loss was less common in younger age groups, with frequency increasing as the patients’ age increased. Hearing loss ranged from 0–11.0% in patients aged 2–17 years across all OI types to 32.1–100% of patients aged ≥50 years (**Table 3**).

Valvulopathies, which included aortic, mitral, pulmonary, and tricuspid valvulopathies, occurred across all OI types, except for patients with Type V OI. The most common type was mitral valvulopathy (**Supplementary Table 5**). For Types I, III, and IV the frequency of valvulopathy increased with age. For Type I OI, valvulopathy increased from 8.3% in patients aged 2–17 years to 15.2% in patients aged ≥50 years; for Type III OI valvulopathy increased from 8.3% in patients aged 2–17 years to 33.3% in patients aged ≥50 years; and from 15.4% to 24.0% in patients with Type IV OI aged 2–17 years and aged 18–49 years, respectively (**Table 3**).

Scleral discoloration, which could be either blue or grey/purple, was recorded in most patients and with bluish sclerae being predominant (**Supplementary Table 6**). The frequency of scleral discoloration ranged from 50% in patients with Type III OI aged 2–17 years to 100% in patients with Type IV OI aged ≥50 years (**Table 3**).

An estimated one-quarter to one-half of all enrolled patients suffered from joint hyperlaxity (**Table 3**). Joint hyperlaxity was typically more common in younger OI patients aged 2–17 years (48.3% of Type I patients, 46.2% of Type IV patients, and 41.4% of patients with unknown type OI).

Dental abnormalities, which included DI and other dental defects, occurred in all patients irrespective of OI type. DI was the most common dental abnormality across patients with Types I, III, IV, and V OI, while patients with unknown OI more frequency had other dental defects (**Supplementary Table 7**). The frequency of dental abnormalities ranged from 7.7% to 50% of patients (patients aged 2–17 years and patients aged ≥50 years with Type IV, respectively).

## 4 Discussion

We previously reported on the skeletal and extraskeletal manifestations in 568 Italian patients with OI (14). Over the previous two years, the data platform was augmented with more detailed clinical features, aiming to provide a more comprehensive overview of each clinical manifestation of the disease. In addition, a re-evaluation of the allocation of specific signs among deformities and limitations was conducted to enhance their relevance to clinical symptoms. Our current study included 628 Italian patients with OI, of which 183 (29.1%) of the patients were new. Finally, this current publication had different data extraction criteria (e.g., slightly different inclusion criteria for the study and the use of baseline [first visit] data compared to the last visit data from our previous publication). To the best of our knowledge, this study is the largest study of OI patients in Italy and provides a comprehensive collection of the natural history data for patients with different types of OI. This study provides insight into how OI affects patients according to their OI type and age. Understanding the natural history of rare diseases, such as OI, help initiatives like the SATURN program, which aims to reduce the time for patient access to life-changing medications (10, 13).

### 4.1 Patient demographics

The demographics of patients enrolled in the study have been previously discussed and were found to be broadly in line with other published studies (1, 14, 16). Despite being evaluated by experienced clinicians and having detailed information on the patient’s clinical characteristics, approximately one-quarter of patients had an unknown OI type – again this is in line with other studies (17, 18). Data on inheritance is in line with literature; most of the pathogenic variants were detected on *COL1A1*, followed by *COL1A2* and then by non-collagen genes, as previously reported by several studies (17, 19, 20). Accurate height prediction of patients with OI enables effective patient monitoring and the development of personalized management plans and, in absence of real-world data, scientists have been investigating the use of machine learning to predict the height curves for patients with OI (21). This is the first large-scale European study to assess the height of adolescent patients with Type I, III, IV, and unknown type of OI over time. Our findings showed that all patients appeared to have a marginally shorter stature compared with the growth standards, when assessed at first measurement, last measurement, and by the annualized evolution of height z-score per patient. Our findings in Type I patients broadly correlate with similar studies conducted in children (aged from birth to 19 years) with OI in Brazil and in patients aged <20 years in North America (22, 23). However, the stature for Type III patients in our study was not as pronounced as other studies, but this is likely the result of data being from a single patient (22, 23).

### 4.2 Fracture frequency

Understanding the fracture rate of patients with OI is essential for their clinical management, evaluating treatment effectiveness, improving patient quality of life, and for evaluating investigative therapies (24). It can also be used as a measurement of disease burden to assist with therapeutic decision-making. A previously published study reported an increase in the number of peripheral fractures with increasing disease severity (1). Our study showed that, over a mean follow-up time of 5.8 years, almost half of patients had at least one fracture, with fractures occurring throughout the body. Patients with Type I OI had the lowest annualized fracture rates, but these were still 2–5-fold higher than the general population (25). Patients with more severe OI types (Type III and Type IV OI) had higher annualized fracture rates than patients with Type I OI, even if for Type III statistical significance was not reached, due to the small number of patients. Similarly, patients with unknown OI type had an increased risk of fractures, compare to Type I. Our study showed a higher annualized fracture rate in children and adolescents and a lower annualized fracture rates in adulthood, which mirrors the findings from a retrospective review of a US claims database, the Danish registry study of fractures in OI patients, and the Australian retrospective chart review study in patients with Type I OI (24, 26, 27). Data from a hospital discharge database in France assessing fractures in pediatric patients with OI showed that ~22.2% of patients reported fractures, which is very similar to our findings (28). We also showed that patients aged ≥50 years had fewer fractures than adult patients aged 18–49 years, which supports the findings of Folkestad et al. (26). Investigations to understand the fracture rate in OI patients suggest that the occurrence of fractures are associated with a bone mineral density z-score of <-2 SD at baseline assessment and the presence of *COL1*-splicing/stop/frameshift variants (29), which may help to explain the differences fracture rates seen in the older populations.

### 4.3 Skeletal manifestations

Patients with OI suffer from a range of skeletal deformities including Wormian bones, kyphoscoliosis, biconcave vertebral bodies, shepherd’s crook femur, anterior tibial bowing, and platybasia (30). Skeletal deformities are directly linked to increased fracture risk, chronic pain, and decreased quality of life in patients with OI (5). Understanding which skeletal deformities occur across the different types of OI and at which ages is crucial for the effective management of patients to improve their quality of life, however data on the frequency of deformities in the different OI types or by age is scarce. We found that skeletal deformities were common across all types of OI and that they increased with increasing age and increasing disease severity. Trunk deformities (which included both spine and rib cage deformities) were the most frequent types of deformity across all OI types, while lower limb deformities were more frequent in the more severe OI types (Type III and IV).

Skeletal abnormalities, including the presence of bone densitometry abnormalities, vertebral compression fractures, Wormian bones, and facial dysmorphisms, are well-known and frequently reported clinical characteristics of OI. However, data on the prevalence of these characteristics by OI type and age is scarce. Here we have reported detailed findings on this much needed data, for example, the presence of Wormian bones is more common in younger ages groups and the more severe OI types (type III and IV) but is not found in older patients. Again, the data on functional limitations by OI type and age is also limited. One study, which focused on motor function and muscle strength in pediatric patients with OI in Brazil, showed that the majority of patients had functional limitations which were exacerbated in patients with Type III OI (31). Another US-based study investigated mobility across OI types and showed that patients with Type I OI typically had minor limitations, but the more severe OI types resulted in more significant mobility limitations (32). We showed that functional limitations occurred in patients with all OI types and ages, with no clear trends. These data show the value of phenotype-specific information to assist with life-course care planning that accommodate adaptations according to disease severity and as the patient ages.

### 4.4 Extraskeletal manifestations

Collagen is a highly abundant protein that is found throughout the body and plays a critical role in maintaining the body’s structural integrity (33). Patients with OI have reduced levels of collagen and, as a result, they can suffer from a range of extraskeletal manifestations. However, little is known about the morbidity and mortality of these patients (16) and, in line with the objectives of the SATURN project (13), information is needed to best understand how to support the management of these patients. What is known, is that the presence of extraskeletal manifestations impacts on the quality of life of patients with OI (16, 34, 35). In absence of real-world data, the use of genetically and phenotypically heterogeneous mouse models of OI have been developed to better understand the potential impact of OI disease-causing genetic variants on extraskeletal manifestations (36).

Our data shows that 73.1% of patients have a scleral abnormality, which corresponds with the high prevalence reported in other studies (1, 18, 27). However, less is known about the impact of this discoloration. Type I collagen is an important structural component of the eye, and it comprises approximately 90% of the sclera. The blue discoloration seen in patients with OI is caused by the thinning of these collagen fibers due to reduced collagen production, thus revealing the underlying choroid (37). As well as acting as a biomarker for OI, a recent review suggests that patients with OI with scleral discoloration are at increased risk of corneal and scleral ruptures after minor trauma. It is recommended, therefore, that patients with OI wear protective glasses and that any ocular surgery is approached with caution (37).

Joint hyperlaxity is a frequent finding in patients with OI and is irrespective of OI type. Hyperlaxity has been linked with skeletal instability and the development scoliosis and can result in asymptomatic or severe craniocervical pathology (38). Our study showed that 39.6% of patients had joint hyperlaxity. Early recognition of patients at risk of developing craniocervical pathology and/or scoliosis is essential for treatment planning.

Our study showed that approximately one-fifth of patients had dental abnormalities. Collagen type I is found in the dentin and periodontal ligament of teeth and lack of collagen can result in dental issues (39). A notable issue for patients with OI is dentinogenesis imperfecta, which has a prevalence of 20–48%, and can cause severe tooth wear and early tooth loss creating speech difficulties, mastication impairment, and challenges for long-term oral health maintenance (39, 40). Again, proactive monitoring of patients with OI with dental issues is essential.

We showed that ~20% of patients with OI suffer from some form of hearing loss, but other reports suggest as many as 70% of patients with OI have hearing impairments. The mechanisms of hearing loss in OI remain unclear and treatments for hearing loss in OI predominantly use conventional treatments for hearing loss, but their efficacy is limited (41). Further work is needed to understand the mechanisms of hearing loss and to develop new approaches for the prevention and management of hearing loss in OI.

More than 70% of skin is collagen type 1, suggesting that patients with OI are more susceptible to skin abnormalities due to their reduced collagen production (36). The percentage of patients with OI who have skin concerns varies with ranges being reported from 15–68% (14, 42); in this study almost one-fifth of patients had skin abnormalities.

It has been previously reported that patients aged ≥50 years have a significant increase in the cumulative incidence of heart failure, mitral and aortic valve regurgitation, and atrial fibrillation and flutter (43). Our study showed that approximately 10% of patients with OI had valvulopathies. As collagen type 1 is an important component of parts of the cardiovascular system, including heart values, our data supports the findings that these patients are at increased risk of cardiovascular disease. Folkestad and colleagues have recommended the closer monitoring of patients with OI after the age of 50 years for cardiac anomalies (43).

Patients with OI typically have a shorter life span than healthy patients, with causes of death being attributed to respiratory diseases, gastrointestinal diseases, and trauma (bone fractures) (44, 45), which are the result of skeletal and extraskeletal manifestations. Understanding the prevalence of these complications will also enable clinicians to provide life-course care planning.

### 4.5 Study limitations

The present study provides a wealth of information to understand the natural history of OI according to type and age. There were, however, some limitations to our study. Firstly, some of the patient groups were comparatively small (e.g., those patients with Type III, IV, and V OI, and some of the numbers of patients in the follow-up studies). As a result, comparisons of data from patients with Type I OI and unknown type OI against data from patients with Type III, IV, and V need to be interpreted with caution – particularly the data around fracture rates by OI. It is possible that these findings may not be representative when applied to larger cohorts of patients with Types III, IV, and V OI. Furthermore, the small sample size for the more severe OI types may also impact longitudinal data. Unfortunately, small numbers of patient groups is a known challenge when working on rare diseases, particularly when evaluating disease types. The number of patients in the different age categories is uneven. While the number of patients in the 2–17 years and 18–49 years groups are broadly comparable, there are fewer patients in the ≥50 years group. Registry-based studies often have a higher proportion of younger patients due to the natural history of these conditions, particularly those that are inherited and manifest in childhood. This is compounded by the fact that specialized care networks actively enroll children, while older patients often find it challenging to find specialist care centers (46). Another limitation is that approximately one-third of patients were classified as unknown type OI as a result of either being in early stages of disease presentation/young children or having clinical manifestations that could be attributed to more than one OI type. In an ideal scenario, each of the patients would undergo genetic testing to confirm their type of OI, but this was outside the scope of this study.

### 4.6 Conclusion

Our study presents the largest cohort of patients with OI from Italy and provides clarity on the natural history of this complex and heterogeneous disease. Our findings show that patients with OI have a stable growth pattern, but typically present with a shorter stature. Patients with OI have an increased annualized fracture rate, which is higher in younger patients and patients with more severe OI types. Patients also present with a range of skeletal and extraskeletal manifestations, some of which may be more common in certain age ranges and OI types. The data from this analysis can be used to support clinicians with the monitoring of their OI patients, including how frequently patients should be assessed and the type of follow-up appointments that would be most beneficial. The ultimate aim of this study is to improve the care of patients with OI.

## Data Availability

The raw data supporting the conclusions of this article will be made available by the authors, upon reasonable request and with the approval of the Ethical Committee.

## Supplementary material

Supplementary Figure 1 Prevalence of skeletal deformities at baseline by OI type and age group among OI patients with follow-up data.

Supplementary Table 1 Prevalence of skeletal manifestations by OI type and age group.

Supplementary Table 2 Prevalence of skeletal manifestations by OI type and age group.

Supplementary Table 3 Prevalence of skin abnormalities by OI type and age group.

Supplementary Table 4 Prevalence of hearing loss by OI type and age group.

Supplementary Table 5 Prevalence of valvulopathy by OI type and age group.

Supplementary Table 6 Prevalence of scleral discoloration by OI type and age group.

Supplementary Table 7 Prevalence of dental defects by OI type and age group.
