# Supplementary material for: Osteogenesis imperfecta: a registry-based study of the clinical symptoms of disease in a large cohort of Italian patients
Source: Front Endocrinol (Lausanne). 2026 May 18;17:1823717. doi: 10.3389/fendo.2026.1823717 (PMC13223775; doi:10.3389/fendo.2026.1823717)
Supplement: Supplementary Figure 1 — Prevalence of skeletal deformities at baseline by OI type and age group among OI patients with follow-up data. [file DataSheet1.doc]

**Supplementary Table 1: Genetic mutations and mode of inheritance by OI type**

| **OI type** | **Type I** | **Type III** | **Type IV** | **Type V** | **Unknown type** | **Overall** |
| --- | --- | --- | --- | --- | --- | --- |
| **Mode of inheritance, n (%)** | | | | | | |
| Negative | 109 (34.9) | 17 (53.1) | 13 (31.0) | 1 (50.0) | 104 (45.0) | 244 (38.9) |
| Non evident* | 10 (3.1) | 2 (6.3) | 0 (0.0) | 0 (0.0) | 12 (5.2) | 24 (3.8) |
| Positive | 140 (43.6) | 4 (12.5) | 20 (47.6) | 0 (0.0) | 87 (37.7) | 251 (40.0) |
| Missing | 62 (19.3) | 9 (28.1) | 9 (21.4) | 1 (50.0) | 28 (12.1) | 109 (17.4) |
| **Mutated gene, n (%)** | | | | | | |
| COL1A1 | 181 (56.4) | 10 (31.3) | 24 (57.1) | 0 (0.0) | 54 (23.4) | 269 (42.8) |
| COL1A2 | 51 (15.9) | 7 (21.9) | 6 (14.3) | 0 (0.0) | 36 (15.6) | 100 (15.9) |
| CRTAP | 0 (0.0) | 1 (3.1) | 0 (0.0) | 0 (0.0) | 0 (0.0) | 1 (0.2) |
| IFITMS | 0 (0.0) | 0 (0.0) | 1 (2.4) | 2 (100) | 0 (0.0) | 3 (0.5) |
| LEPRE1 | 0 (0.0) | 2 (6.3) | 0 (0.0) | 0 (0.0) | 1 (0.4) | 3 (0.5) |
| SERPINF1 | 0 (0.0) | 0 (0.0) | 0 (0.0) | 0 (0.0) | 3 (1.3) | 3 (0.5) |
| WNT1 | 0 (0.0) | 0 (0.0) | 0 (0.0) | 0 (0.0) | 1 (0.4) | 1 (0.2) |
| Missing | 89 (27.7) | 12 (37.5) | 11 (26.2) | 0 (0.0) | 136 (58.9) | 248 (39.5) |

*Inheritance was classified as ‘Non evident’ when it could not be determined if the patient’s OI was inherited from a family member.

**Supplementary Figure 1: Prevalence of skeletal deformities by OI type and age group at baseline – in patients with at least 2 data points more than 1 year apart**

Legend: Patients may have more than one deformity type. Error bars represent the 95% confidence intervals. Confidence intervals were displayed only for prevalence measures >0% and <100%.

**Supplementary Table 2: Skeletal manifestations by OI type and age**

| **Skeletal manifestations** | **Bone densitometry abnormality*** | **Vertebral compression fractures** | **Wormian bones** | **Facial dysmorphisms^†^** | **Functional limitations^‡^** |
| --- | --- | --- | --- | --- | --- |
| **Type I OI** | | | | | |
| 2–17 years (n=145), n (%) [95% CI] | 64 (44.1)  [35.9, 52.6] | 3 (2.1)  [0.4, 5.9] | 14 (9.6)  [5.4, 15.7] | 21 (14.5)  [9.2, 21.3] | 16 (11.0)  [6.4, 17.3] |
| 18–49 years (n=143), n (%) [95% CI] | 80 (55.9)  [47.4, 64.2] | 6 (4.2)  [1.6, 8.9] | 5 (3.5)  [1.1, 8.0] | 19 (13.3)  [8.2, 20.0] | 22 (15.4)  [9.9, 22.4] |
| ≥50 years (n=33), n (%) [95% CI] | 22 (66.7)  [48.2, 82.0] | 3 (9.1)  [1.9, 24.3] | 0 (0.0)  [0.0, 10.6] | 1 (3.0)  [0.1, 15.8] | 8 (24.2)  [11.1, 42.3] |
| **Type III OI** | | | | | |
| 2–17 years (n=12), n (%) [95% CI] | 6 (50%)  [21.1, 78.9] | 1 (8.3)  [0.2, 38.5] | 2 (16.7)  [2.1, 48.4] | 4 (33.3)  [9.9, 65.1] | 0 (0.0)  [0.0, 26.5] |
| 18–49 years (n=17), n (%) [95% CI] | 8 (47.1)  [23.0, 72.2] | 0 (0.0)  [0.0, 19.5] | 4 (23.5)  [6.8, 49.9] | 10 (58.8)  [32.9, 81.6] | 2 (11.8)  [1.5, 36.4] |
| ≥50 years (n=3), n (%) [95% CI] | 1 (33.3)  [0.8, 90.6] | 0 (0.0)  [0.0, 70.8] | 0 (0.0)  [0.0, 70.8] | 1 (33.3)  [0.8, 90.6] | 1 (33.3)  [0.8, 90.6] |
| **Type IV OI** | | | | | |
| 2–17 years (n=13), n (%) [95% CI] | 7 (53.8)  [25.1, 80.8] | 1 (7.7)  [0.2, 36.0] | 3 (23.1)  [5.0, 53.8] | 5 (38.5)  [13.9, 68.4] | 2 (15.4)  [1.9, 45.4] |
| 18–49 years (n=25), n (%) [95% CI] | 15 (60.0)  [38.7, 78.9] | 1 (4.0)  [0.1, 20.4] | 0 (0.0)  [0.0, 13.7] | 11 (44.0)  [24.4, 65.1] | 3 (12.0)  [2.5, 31.2] |
| ≥50 years (n=4), n (%) [95% CI] | 3 (75.0)  [19.4, 99.4] | 1 (25.0)  [0.6, 80.6] | 0 (0.0)  [0.0, 60.2] | 1 (25.0)  [0.6, 80.6] | 0 (0.0)  [0.0, 60.2] |
| **Type V OI** | | | | | |
| 2–17 years (n=1), n (%) [95% CI] | 1 (100)  [2.5, 100.0] | 0 (0.0)  [0.0, 97.5] | 1 (100)  [2.5, 100.0] | 0 (0.0)  [0.0, 97.5] | 0 (0.0)  [0.0, 97.5] |
| 18–49 years (n=1), n (%) [95% CI] | 1 (100.0)  [2.5, 100.0] | 0 (0.0)  [0.0, 97.5] | 0 (0.0)  [0.0, 97.5] | 1 (100.0)  [2.5, 100.0] | 1 (100.0)  [2.5, 100.0] |
| ≥50 years (n=0), n (%) [95% CI] | NC | NC | NC | NC | NC |
| **Unknown type OI** | | | | | |
| 2–17 years (n=116), n (%) [95% CI] | 32 (27.6)  [19.7, 36.7] | 5 (4.3)  [1.4, 9.8] | 3 (2.6)  [0.5, 7.4] | 21 (18.1)  [11.6, 26.3] | 21 (18.1)  [11.6, 26.3] |
| 18–49 years (n=87), n (%) [95% CI] | 53 (60.9)  [49.9, 71.2] | 12 (13.8)  [7.3, 22.9] | 0 (0.0)  [0.0, 4.2] | 5 (5.7)  [1.9, 12.9] | 17 (19.5)  [11.8, 29.4] |
| ≥50 years (n=28), n (%) [95% CI] | 22 (78.6)  [59.0, 91.7] | 7 (25.0)  [10.7, 44.9] | 0 (0.0)  [0.0, 12.3] | 7 (25.0)  [10.7, 44.9] | 5 (17.9)  [6.1, 36.9] |

Legend: *Includes osteopenia and osteoporosis. ^†^Includes triangular face, maxilla dysmorphism, frontal bossing, and other facial dysmorphisms. ^‡^Includes limitations of the head and neck, upper limbs, lower limbs, trunk, and hip and pelvis. CI, confidence interval; NC, not calculated due to absence of patients.

**Supplementary Table 3: Skin abnormalities by OI type and age**

| **Extraskeletal manifestations: Skin abnormalities*** | **Any skin abnormality** | **Morphological skin abnormality** | **Cutis laxa** | **Skin lesion** | **Other skin abnormality** |
| --- | --- | --- | --- | --- | --- |
| **Type I OI** | | | | | |
| 2–17 years (n=145), n % [95% CI] | 25 (17.1)  [11.5, 24.4] | 7 (4.8)  [2.0, 9.7] | 14 (9.6)  [5.4, 15.7] | 6 (4.2)  [1.5, 8.8] | 0 (0.0)  [0.0, 2.5] |
| 18–49 years (n=143), n % [95% CI] | 27 (18.9)  [12.8, 26.3] | 6 (4.2)  [1.6, 8.9] | 14 (9.8)  [5.5, 15.9] | 10 (7.0)  [3.4, 12.5] | 1 (0.7)  [0.0, 3.8] |
| ≥50 years (n=33), n % [95% CI] | 9 (27.3) [13.3, 45.5] | 3 (9.1)  [1.9, 24.3] | 6 (18.2)  [7.0, 35.5] | 0 (0.0)  [0.0, 10.6] | 0 (0.0)  [0.0, 10.6] |
| **Type III OI** | | | | | |
| 2–17 years (n=12), n % [95% CI] | 3 (25.0)  [5.5, 57.2] | 1 (8.3)  [0.2, 38.5] | 2 (16.7)  [2.1, 48.4] | 0 (0.0)  [0.0, 26.5] | 0 (0.0)  [0.0, 26.5] |
| 18–49 years (n=17), n % [95% CI] | 2 (11.2)  [1.5, 36.4] | 1 (5.9)  [0.1, 28.7] | 1 (5.9)  [0.1, 28.7] | 0 (0.0)  [0.0, 19.5] | 0 (0.0)  [0.0, 19.5] |
| ≥50 years (n=3), n % [95% CI] | 0 (0.0)  [0.0, 70.8] | 0 (0.0)  [0.0, 70.8] | 0 (0.0)  [0.0, 70.8] | 0 (0.0)  [0.0, 70.8] | 0 (0.0)  [0.0, 70.8] |
| **Type IV OI** | | | | | |
| 2–17 years (n=13), n % [95% CI] | 2 (15.4)  [1.9, 45.4] | 0 (0.0)  [0.0, 24.7] | 2 (15.4)  [1.9, 45.4] | 0 (0.0)  [0.0, 24.7] | 0 (0.0)  [0.0, 24.7] |
| 18–49 years (n=25), n % [95% CI] | 3 (12.0)  [2.5, 31.2] | 1 (4.0)  [0.1, 20.4] | 2 (8.0)  [1.0, 26.0] | 0 (0.0)  [0.0, 13.7] | 0 (0.0)  [0.0, 13.7] |
| ≥50 years (n=4), n % [95% CI] | 1 (25.0)  [0.6, 80.6] | 0 (0.0)  [0.0, 60.2] | 1 (25.0)  [0.6, 80.6] | 0 (0.0)  [0.0, 60.2] | 0 (0.0)  [0.0, 60.2] |
| **Type V OI** | | | | | |
| 2–17 years (n=1), n % [95% CI] | 0 (0.0)  [0.0, 97.5] | 0 (0.0)  [0.0, 97.5] | 0 (0.0)  [0.0, 97.5] | 0 (0.0)  [0.0, 97.5] | 0 (0.0)  [0.0, 97.5] |
| 18–49 years (n=1), n % [95% CI] | 1 (100.0)  [2.5, 100.0] | 0 (0.0)  [0.0, 97.5] | 1 (100.0)  [2.5, 100.0] | 0 (0.0)  [0.0, 97.5] | 0 (0.0)  [0.0, 97.5] |
| ≥50 years (n=0), n % [95% CI] | NC | NC | NC | NC | NC |
| **Unknown type OI** | | | | | |
| 2–17 years (n=116), n % [95% CI] | 21 (18.1)  [11.6, 26.3] | 1 (0.9)  [0.0, 4.7] | 14 (12.1)  [6.8, 19.4] | 7 (6.0)  [2.5, 12.0] | 0 (0.0)  [0.0, 3.1] |
| 18–49 years (n=87), n % [95% CI] | 8 (9.2)  [4.1, 17.3] | 0 (0.0)  [0.0, 4.2] | 4 (4.6)  [1.3, 11.4] | 2 (2.3)  [0.3, 8.1] | 2 (2.3)  [0.3, 8.1] |
| ≥50 years (n=28), n % [95% CI] | 6 (21.4)  [8.3, 41.0] | 1 (3.6)  [0.1, 18.3] | 4 (14.3)  [4.0, 32.7] | 1 (3.6)  [0.1, 18.3] | 0 (0.0)  [0.0, 12.3] |

Legend: *Patients may have more than one type of skin abnormality. CI, confidence interval; NC, not calculated due to absence of patients.

**Supplementary Table 4: Hearing loss by OI type and age**

| **Extraskeletal manifestations: Hearing loss** | **Any hearing loss** | **Mixed hearing loss** | **Conductive hearing loss** | **Sensorineural hearing loss** | **Other hearing loss** |
| --- | --- | --- | --- | --- | --- |
| **Type I OI** | | | | | |
| 2–17 years (n=145), n % [95% CI] | 16 (11.0)  [6.4, 17.3] | 4 (2.7)  [0.8, 6.9] | 8 (5.5)  [2.4, 10.6] | 4 (2.7)  [0.8, 6.9] | 0 (0.0)  [0.0, 2.5] |
| 18–49 years (n=143), n % [95% CI] | 36 (25.2)  [18.3, 33.1] | 5 (3.5)  [1.1, 8.0] | 11 (7.7)  [3.9, 13.3] | 12 (8.4)  [4.4, 14.2] | 8 (5.6)  [2.4, 10.7] |
| ≥50 years (n=33), n % [95% CI] | 18 (54.5)  [36.4, 71.9] | 2 (6.1)  [0.7, 20.2] | 5 (15.2)  [5.1, 31.9] | 7 (21.2)  [9.0, 38.9] | 4 (12.1)  [3.4, 28.2] |
| **Type III OI** | | | | | |
| 2–17 years (n=12), n % [95% CI] | 1 (8.3)  [0.2, 38.5] | 0 (0.0)  [0.0, 26.5] | 1 (8.3)  [0.2, 38.5] | 0 (0.0)  [0.0, 26.5] | 0 (0.0)  [0.0, 26.5] |
| 18–49 years (n=17), n % [95% CI] | 4 (23.5)  [6.8, 49.9] | 2 (11.8)  [1.5, 36.4] | 1 (5.9)  [0.1, 28.7] | 1 (5.9)  [0.1, 28.7] | 0 (0.0)  [0.0, 19.5] |
| ≥50 years (n=3), n % [95% CI] | 3 (100.0)  [29.2, 100.0] | 0 (0.0)  [0.0, 70.8] | 0 (0.0)  [0.0, 70.8] | 2 (66.7)  [9.4, 99.2] | 1 (33.3)  [0.8, 90.6] |
| **Type IV OI** | | | | | |
| 2–17 years (n=13), n % [95% CI] | 0 (0.0)  [0.0, 24.7] | 0 (0.0)  [0.0, 24.7] | 0 (0.0)  [0.0, 24.7] | 0 (0.0)  [0.0, 24.7] | 0 (0.0)  [0.0, 24.7] |
| 18–49 years (n=25), n % [95% CI] | 7 (28.0)  [12.1, 49.4] | 2 (8.0)  [1.0, 26.0] | 1 (4.0)  [0.1, 20.4] | 3 (12.0)  [2.5, 31.2] | 1 (4.0)  [0.1, 20.4] |
| ≥50 years (n=4), n % [95% CI] | 2 (50.0)  [6.8, 93.2] | 0 (0.0)  [0.0, 60.2] | 0 (0.0)  [0.0, 60.2] | 2 (50.0)  [6.8, 93.2] | 0 (0.0)  [0.0, 60.2] |
| **Type V OI** | | | | | |
| 2–17 years (n=1), n % [95% CI] | 0 (0.0)  [0.0, 97.5] | 0 (0.0)  [0.0, 97.5] | 0 (0.0)  [0.0, 97.5] | 0 (0.0)  [0.0, 97.5] | 0 (0.0)  [0.0, 97.5] |
| 18–49 years (n=1), n % [95% CI] | 0 (0.0)  [0.0, 97.5] | 0 (0.0)  [0.0, 97.5] | 0 (0.0)  [0.0, 97.5] | 0 (0.0)  [0.0, 97.5] | 0 (0.0)  [0.0, 97.5] |
| ≥50 years (n=0), n % [95% CI] | NC | NC | NC | NC | NC |
| **Unknown type OI** | | | | | |
| 2–17 years (n=116), n % [95% CI] | 2 (1.7)  [0.2, 6.1] | 1 (0.9)  [0.0, 4.7] | 0 (0.0)  [0.0, 3.1] | 0 (0.0)  [0.0, 3.1] | 1 (0.9)  [0.0, 4.7] |
| 18–49 years (n=87), n % [95% CI] | 15 (17.2)  [10.0, 26.8] | 2 (2.3)  [0.3, 8.1] | 6 (6.9)  [2.6, 14.4] | 4 (4.6)  [1.3, 11.4] | 3 (3.4)  [0.7, 9.7] |
| ≥50 years (n=28), n % [95% CI] | 9 (32.1)  [15.9, 52.4] | 0 (0.0)  [0.0, 12.3] | 2 (7.1)  [0.9, 23.5] | 2 (7.1)  [0.9, 23.5] | 5 (17.9)  [6.1, 36.9] |

Legend: CI, confidence interval; NC, not calculated due to absence of patients.

**Supplementary Table 5: Valvulopathy by OI type and age**

| **Extraskeletal manifestations: Valvulopathy*** | **Any valvulopathy** | **Aortic valvulopathy** | **Mitral valvulopathy** | **Pulmonary valvulopathy** | **Tricuspid valvulopathy** |
| --- | --- | --- | --- | --- | --- |
| **Type I OI** | | | | | |
| 2–17 years (n=145), n % [95% CI] | 12 (8.2)  [4.3, 14.0] | 1 (0.7)  [0.0, 3.8] | 7 (4.8)  [2.0, 9.7] | 3 (2.1)  [0.4, 5.9] | 3 (2.1)  [0.4, 5.9] |
| 18–49 years (n=143), n % [95% CI] | 14 (9.8)  [5.5, 15.9] | 1 (0.7)  [0.0, 3.8] | 8 (5.6)  [2.4, 10.7] | 2 (1.4)  [0.2, 5.0] | 4 (2.8)  [0.8, 7.0] |
| ≥50 years (n=33), n % [95% CI] | 5 (15.2)  [5.1, 31.9] | 2 (6.1)  [0.7, 20.2] | 3 (9.1)  [1.9, 24.3] | 0 (0.0)  [0.0, 10.6] | 2 (6.1)  [0.7, 20.2] |
| **Type III OI** | | | | | |
| 2–17 years (n=12), n % [95% CI] | 1 (8.3)  [0.2, 38.5] | 0 (0.0)  [0.0, 26.5] | 0 (0.0)  [0.0, 26.5] | 0 (0.0)  [0.0, 26.5] | 1 (8.3)  [0.2, 38.5] |
| 18–49 years (n=17), n % [95% CI] | 1 (5.9)  [0.1, 28.7] | 0 (0.0)  [0.0, 19.5] | 1 (5.9)  [0.1, 28.7] | 0 (0.0)  [0.0, 19.5] | 1 (5.9)  [0.1, 28.7] |
| ≥50 years (n=3), n % [95% CI] | 1 (33.3)  [0.8, 90.6] | 0 (0.0)  [0.0, 70.8] | 1 (33.3)  [0.8, 90.6] | 0 (0.0)  [0.0, 70.8] | 0 (0.0)  [0.0, 70.8] |
| **Type IV OI** | | | | | |
| 2–17 years (n=13), n % [95% CI] | 2 (15.4)  [1.9, 45.4] | 1 (7.7)  [0.2, 36.0] | 0 (0.0)  [0.0, 24.7] | 0 (0.0)  [0.0, 24.7] | 1 (7.7)  [0.2, 36.0] |
| 18–49 years (n=25), n % [95% CI] | 6 (24.0)  [9.4, 45.1] | 2 (8.0)  [1.0, 26.0] | 3 (12.0)  [2.5, 31.2] | 1 (4.0)  [0.1, 20.4] | 1 (4.0)  [0.1, 20.4] |
| ≥50 years (n=4), n % [95% CI] | 0 (0.0)  [0.0, 60.2] | 0 (0.0)  [0.0, 60.2] | 0 (0.0)  [0.0, 60.2] | 0 (0.0)  [0.0, 60.2] | 0 (0.0)  [0.0, 60.2] |
| **Type V OI** | | | | | |
| 2–17 years (n=1), n % [95% CI] | 0 (0.0)  [0.0, 97.5] | 0 (0.0)  [0.0, 97.5] | 0 (0.0)  [0.0, 97.5] | 0 (0.0)  [0.0, 97.5] | 0 (0.0)  [0.0, 97.5] |
| 18–49 years (n=1), n % [95% CI] | 0 (0.0)  [0.0, 97.5] | 0 (0.0)  [0.0, 97.5] | 0 (0.0)  [0.0, 97.5] | 0 (0.0)  [0.0, 97.5] | 0 (0.0)  [0.0, 97.5] |
| ≥50 years (n=0), n % [95% CI] | NC | NC | NC | NC | NC |
| **Unknown type OI** | | | | | |
| 2–17 years (n=116), n % [95% CI] | 8 (6.9)  [3.0, 13.1] | 2 (1.7)  [0.2, 6.1] | 5 (4.3)  [1.4, 9.8] | 0 (0.0)  [0.0, 3.1] | 1 (0.9)  [0.0, 4.7] |
| 18–49 years (n=87), n % [95% CI] | 8 (9.2)  [4.1, 17.3] | 0 (0.0)  [0.0, 4.2] | 8 (9.2)  [4.1, 17.3] | 0 (0.0)  [0.0, 4.2] | 0 (0.0)  [0.0, 4.2] |
| ≥50 years (n=28), n % [95% CI] | 1 (3.6)  [0.1, 18.3] | 0 (0.0)  [0.0, 12.3] | 1 (3.6)  [0.1, 18.3] | 0 (0.0)  [0.0, 12.3] | 0 (0.0)  [0.0, 12.3] |

Legend: *Patients may have more than one type of valvulopathy. CI, confidence interval; NC, not calculated due to absence of patients.

**Supplementary Table 6: Scleral discoloration by OI type and age**

| **Extraskeletal manifestations: Scleral discoloration** | **Any scleral discoloration** | **Blue** | **Grey/purple** |
| --- | --- | --- | --- |
| **Type I OI** | | | |
| 2–17 years (n=145), n %  [95% CI] | 126 (86.3)  [81.6, 92.9] | 103 (70.5)  [63.9, 79.2] | 23 (15.8)  [10.5, 23.1] |
| 18–49 years (n=143), n %  [95% CI] | 121 (84.6)  [77.6, 90.1] | 104 (72.7)  [64.7, 79.8] | 17 (11.9)  [7.1, 18.4] |
| ≥50 years (n=33), n %  [95% CI] | 25 (75.8)  [57.7, 88.9] | 21 (63.6)  [45.1, 79.6] | 4 (12.1)  [3.4, 28.2] |
| **Type III OI** | | | |
| 2–17 years (n=12), n %  [95% CI] | 6 (50.0)  [21.1, 78.9] | 5 (41.7)  [15.2, 72.3] | 1 (8.3)  [0.2, 38.5] |
| 18–49 years (n=17), n %  [95% CI] | 13 (76.5)  [50.1, 93.2] | 8 (47.1)  [23.0, 72.2] | 5 (29.4)  [10.3, 56.0] |
| ≥50 years (n=3), n %  [95% CI] | 2 (66.7)  [9.4, 99.2] | 2 (66.7)  [9.4, 99.2] | 0 (0.0)  [0.0, 70.8] |
| **Type IV OI** | | | |
| 2–17 years (n=13), n %  [95% CI] | 12 (92.3)  [64.0, 99.8] | 10 (76.9)  [46.2, 95.0] | 2 (15.4)  [1.9, 45.4] |
| 18–49 years (n=25), n %  [95% CI] | 20 (80.0)  [59.9, 93.2] | 14 (53.8)  [34.9, 75.6] | 6 (24.0)  [9.4, 45.1] |
| ≥50 years (n=4), n %  [95% CI] | 4 (100.0)  [39.8, 100.0] | 4 (100.0)  [39.8, 100.0] | 0 (0.0)  [0.0, 60.2] |
| **Type V OI** | | | |
| 2–17 years (n=1), n %  [95% CI] | 0 (0.0)  [0.0, 97.5] | 0 (0.0)  [0.0, 97.5] | 0 (0.0)  [0.0, 97.5] |
| 18–49 years (n=1), n %  [95% CI] | 0 (0.0)  [0.0, 97.5] | 0 (0.0)  [0.0, 97.5] | 0 (0.0)  [0.0, 97.5] |
| ≥50 years (n=0), n %  [95% CI] | NC | NC | NC |
| **Unknown type OI** | | | |
| 2–17 years (n=116), n % [95% CI] | 66 (56.9)  [47.4, 66.1] | 52 (44.8)  [35.6, 54.3] | 14 (12.1)  [6.8, 19.4] |
| 18–49 years (n=87), n % [95% CI] | 45 (51.7)  [40.8, 62.6] | 41 (47.1)  [36.3, 58.1] | 4 (4.6)  [1.3, 11.4] |
| ≥50 years (n=28), n %  [95% CI] | 19 (67.9)  [47.6, 84.1] | 17 (60.7)  [40.6, 78.5] | 2 (7.1)  [0.9, 23.5] |

Legend: CI, confidence interval; NC, not calculated due to absence of patients.

**Supplementary Table 7: Dental defects by OI type and age**

| **Extraskeletal manifestations: Dental defect*** | **Any dental defect** | **Dentinogenesis imperfecta** | **Other dental defect** |
| --- | --- | --- | --- |
| **Type I OI** | | | |
| 2–17 years (n=145), n %  [95% CI] | 14 (9.6)  [5.5, 15.9] | 8 (5.5)  [2.4, 10.7] | 6 (4.1)  [1.6, 8.9] |
| 18–49 years (n=143), n %  [95% CI] | 28 (19.6)  [13.4, 27.0] | 20 (14.0)  [8.8, 20.8] | 8 (5.6)  [2.4, 10.7] |
| ≥50 years (n=33), n %  [95% CI] | 9 (27.3)  [13.3, 45.5] | 4 (12.1)  [3.4, 28.2] | 5 (15.2)  [5.1, 31.9] |
| **Type III OI** | | | |
| 2–17 years (n=12), n %  [95% CI] | 3 (25.0)  [5.5, 57.2] | 3 (25.0)  [5.5, 57.2] | 0 (0.0)  [0.0, 26.5] |
| 18–49 years (n=17), n %  [95% CI] | 8 (47.1)  [23.0, 72.2] | 8 (47.1)  [23.0, 72.2] | 0 (0.0)  [0.0, 19.5] |
| ≥50 years (n=3), n %  [95% CI] | 1 (33.3)  [0.8, 90.6] | 1 (33.3)  [0.8, 90.6] | 0 (0.0)  [0.0, 70.8] |
| **Type IV OI** | | | |
| 2–17 years (n=13), n %  [95% CI] | 1 (7.7)  [0.2, 36.0] | 1 (7.7)  [0.2, 36.0] | 0 (0.0)  [0.0, 24.7] |
| 18–49 years (n=25), n %  [95% CI] | 11 (44.0)  [24.4, 65.1] | 11 (44.0)  [24.4, 65.1] | 0 (0.0)  [0.0, 13.7] |
| ≥50 years (n=4), n %  [95% CI] | 2 (50.0)  [6.8, 93.2] | 0 (0.0)  [0.0, 60.2] | 2 (50.0)  [6.8, 93.2] |
| **Type V OI** | | | |
| 2–17 years (n=1), n %  [95% CI] | 0 (0.0)  [0.0, 97.5] | 0 (0.0)  [0.0, 97.5] | 0 (0.0)  [0.0, 97.5] |
| 18–49 years (n=1), n %  [95% CI] | 0 (0.0)  [0.0, 97.5] | 0 (0.0)  [0.0, 97.5] | 0 (0.0)  [0.0, 97.5] |
| ≥50 years (n=0), n %  [95% CI] | NC | NC | NC |
| **Unknown type OI** | | | |
| 2–17 years (n=116), n %  [95% CI] | 22 (19.0)  [12.3, 27.3] | 10 (8.6)  [4.2, 15.3] | 12 (10.3)  [5.5, 17.4] |
| 18–49 years (n=87), n %  [95% CI] | 19 (21.8)  [13.7, 32.0] | 8 (9.2)  [4.1, 17.3] | 11 (12.6)  [6.5, 21.5] |
| ≥50 years (n=28), n %  [95% CI] | 5 (17.9)  [6.1, 36.9] | 2 (7.1)  [0.9, 23.5] | 3 (10.7)  [2.3, 28.2] |

Legend: *Patients may have more than one type of dental defect. CI, confidence interval; NC, not calculated due to absence of patients.
